# Signing at the beginning versus at the end does not decrease dishonesty

**DOI:** 10.1073/pnas.1911695117

**Published:** 2020-03-31

**Authors:** Ariella S. Kristal, Ashley V. Whillans, Max H. Bazerman, Francesca Gino, Lisa L. Shu, Nina Mazar, Dan Ariely

**Affiliations:** ^a^Harvard Business School, Harvard University, Boston, MA 02163;; ^b^Organisational Behaviour, London Business School, London, United Kingdom NW1 4SA;; ^c^Questrom School of Business, Boston University, Boston, MA 02215;; ^d^Fuqua School of Business, Duke University, Durham, NC 27708

**Keywords:** nudge, policy-making, morality, replication

## Abstract

Honest reporting is essential for society to function well. However, people frequently lie when asked to provide information, such as misrepresenting their income to save money on taxes. A landmark finding published in PNAS [L. L. Shu, N. Mazar, F. Gino, D. Ariely, M. H. Bazerman, *Proc. Natl. Acad. Sci. U.S.A.* 109, 15197–15200 (2012)] provided evidence for a simple way of encouraging honest reporting: asking people to sign a veracity statement at the beginning instead of at the end of a self-report form. Since this finding was published, various government agencies have adopted this practice. However, in this project, we failed to replicate this result. Across five conceptual replications (*n* = 4,559) and one highly powered, preregistered, direct replication (*n* = 1,235) conducted with the authors of the original paper, we observed no effect of signing first on honest reporting. Given the policy applications of this result, it is important to update the scientific record regarding the veracity of these results.

## Significance

In 2012, five of the current authors published a paper in PNAS showing that people are more honest when they are asked to sign a veracity statement at the beginning instead of at the end of a tax or insurance audit form. In a recent investigation, across five related experiments we failed to find an effect of signing at the beginning on dishonesty. Following up on these studies, we conducted one preregistered, high-powered direct replication of experiment 1 of the PNAS paper, in which we failed to replicate the original result. The current paper updates the scientific record by showing that signing at the beginning is unlikely to be a simple solution for increasing honest reporting.

Five of the seven authors of this manuscript conducted research published in PNAS (1), showing that signing a veracity statement at the beginning of a tax form (in two small-sample laboratory studies) as well as an insurance audit form (in a field experiment), as opposed to the standard procedure of signing it at the end of the form, decreases dishonest reporting of personal information. The original paper also found that signing first reduces dishonesty by making ethics more salient, although this effect was demonstrated only in one small-sample laboratory experiment. Three of the authors (Kristal, Whillans, and Bazerman) attempted to extend what we thought was the critical finding from that PNAS paper: simply signing a veracity statement at the beginning instead of at the end of a task in which individuals can cheat and report a higher performance to earn more money reduces dishonest reporting. In particular, Kristal, Whillans, and Bazerman initiated a new project on inducing honesty online by signing first versus last. However, despite repeated attempts, Kristal, Whillans, and Bazerman did not find that signing a veracity statement at the beginning of a task reduced dishonesty in comparison to signing at the end. As a consequence of these null effects, together with the original authors of the PNAS paper, the authors then set out to conduct a direct replication of the first laboratory experiment described in the PNAS paper (1).

There are several reasons why Kristal, Whillans, and Bazerman continued to pursue this line of research and why we eventually conducted a direct replication. The original finding has been cited 345 times in peer-reviewed publications, and governments around the world have spent time and effort operationalizing this finding in various policy domains. We are also aware of two published failures of signing first to increase tax collection. The most relevant is an experiment that was conducted in a local authority in the United Kingdom (2). In this experiment, the intervention directly tested the veracity statement and signature requirement at the top versus at the bottom with no significant differences. A second relevant experiment was conducted in Guatemala (3), but in this case the test compared adding an additional signature prompt before filling out a tax form compared with no additional prompt. The additional signature prompt was part of a CAPTCHA pop-up box window and not of the tax form itself (but there was a signature box at the end of the form in both conditions). There is one reported success in the United States (at increasing median amount of sales declared by goods and services vendors to the government, with no further statistical information provided). Yet, this intervention did not directly test the sign first versus last mechanism and instead added a signature prompt at the beginning of a form compared with the standard procedure of having no signature prompt (4). However, field experiments are often conducted in noisy environments, allowing many alternative explanations that could account for potentially mixed results. Thus, tightly controlled laboratory experiments are needed to confirm the conclusion of these studies.

The hypothesis that was tested in the original PNAS paper was that signing a veracity statement at the beginning (vs. the standard procedure of signing at the end) of a self-report form like a tax or an insurance audit form would reduce dishonest reporting by making ethics more salient before someone had the opportunity to cheat. To test this hypothesis in the original PNAS paper (1), there were two laboratory experiments (*n* = 101 and *n* = 60, respectively) and one field experiment (*n* = 13,488). Across the two laboratory experiments, participants were asked to self-report on a tax form their income from a previously completed task and their travel expenses incurred to participate in the study, and they were paid according to their self-reports. In experiment 1, one-third of the participants were asked to sign a veracity statement “I declare that I carefully examined this return and that to the best of my knowledge and belief it is correct and complete” that was placed at the beginning of the tax form before reporting the two amounts, while another third saw the veracity statement at the end of the tax form after reporting the two amounts, and the final third did not see a veracity statement (control). In experiment 2 we only had the two conditions—before versus after reporting. Unbeknownst to participants, the authors knew exactly how much of the task participants accurately completed, allowing authors to assess cheating—as measured by the percentage of people who cheated through overreporting their income, as well the specific amounts they overclaimed. Consistent with the stated hypothesis, in these two laboratory studies (1), fewer participants cheated (2), they claimed more accurate performances, and (3) claimed fewer expenses (likely due to more honest reporting of their expenses) when the veracity statement that they were asked to sign was placed at the top of the tax form (vs. at the bottom).

The original PNAS paper also replicated these findings in a real-world insurance setting, where customers were purportedly randomly assigned to report the odometer reading of their cars on an audit form sent by their automobile insurance company, and they encountered and were asked to sign a veracity statement either at the bottom (standard practice) or at the top of filling out the form. Based on the odometer reading reported on the previous audit form we then calculated customers’ reported use of their car (i.e., number of miles driven). However, the reported odometer readings in the two conditions were significantly different at baseline.

One possible interpretation of the baseline difference in reported miles driven is that the randomization failed (or may have even failed to occur as instructed) in that study. The significant findings in terms of difference in reported miles holds when controlling for miles driven at baseline. See Table 1 for summary statistics from the original field experiment. The original field data are now available at https://osf.io/3javq/ along with the rest of the materials for this paper.

**Table 1.** Summary statistics from study 3 from Shu et al. (1)

	Sign-at-the-bottom, means (SD)	Sign-at-the-top, means (SD)	Two-sided *t* test, values
Baseline odometer reading (*t*0)	75,034.50 (50,265.35)	59,692.71 (49,953.51)	*t*_(13,474)_ = 17.78, *P* < 0.0001
New odometer reading (*t*1)	98,705.14 (51,934.76)	85,791.10 (51,701.31)	*t*_(13,475)_ = 14.47, *P* < 0.0001
Difference in odometer readings; i.e., miles driven (*t*1–*t*0)*	23,670.64 (12,621.38)	26,098.40 (12,253.37)	*t*_(13,448)_ = −11.331, *P* < 0.0001

* This row was the outcome reported in the original paper.

As Kristal, Whillans, and Bazerman launched their investigation of inducing honesty online, they conducted a series of laboratory experiments in which they asked participants to sign a veracity statement before (vs. after) reporting their performance on a task where the pay was tied to their performance (“sign-first”). Across their first four studies, they failed to obtain a significant result, either in an online or an offline context.

As they obtained repeated failures to find a significant sign-first effect in both online and offline contexts, they shifted the focus of their project to replicate the original result and asked all original authors to join the project for study 6.

Studies 1–5 were conducted as part of a larger project examining whether people were more likely to cheat in an online (vs. offline environment).*

In study 1, participants were told to roll dice and report the total of their roll in exchange for lottery tickets. Participants were randomly assigned to a sign-first condition (before reporting), a sign-last condition, or a control condition where participants were not asked to provide signatures (see *Materials and Methods* for more details). Given the design of the experiment, it was not possible to tell what participants’ individual true performances were, but the average number reported could be compared both to the expected distribution under fair conditions and to each of the conditions.

In studies 2–5, participants were asked to complete a series of tasks that allowed us to examine individual-level cheating. In these four studies, participants were randomly assigned to complete a veracity statement that was placed before or after reporting how much of the task they completed. All tasks included unsolvable items. Cheating was defined as people reporting having completed more “solved” items than the possible maximum solvable (i.e., participants’ true performance was somewhat accessible).

Studies 1–5 were intended to study whether online cheating could be reduced by asking people to provide an online signature before versus after reporting, rather than the goal of providing a replication, and as a result, these studies did not include the identical methods that were originally used in the PNAS paper (1). One possibility is that differences in methods could have potentially accounted for the null results that Kristal, Whillans, and Bazerman observed in studies 1–5; specifically, while all studies were incentive-compatible, these new studies used forms where there was not necessarily an established norm for providing a signature at the bottom and no cost for dishonesty, whereas the Shu et al. (1) experiments included tax and audit forms where people typically expect to provide their signature at the end and would risk punishment for dishonesty. Thus, in study 6, we conducted a direct replication of study 1 of the PNAS (1) manuscript (leaving out the pure control condition: no signature), given that it was the most tightly controlled laboratory experiment and it produced the largest effect size for percent of people overclaiming their performance income (*d* = 0.70) (1). We conducted this study using current best practices for ensuring replicable research (5): we conducted adequate power calculations before undertaking the direct replication, we preregistered our analyses, and we made all of our materials publicly available.

## Results

In Table 2, we report the sample sizes, tasks, populations, and confidence intervals of the studies in the original paper and the current paper. Whereas in the original PNAS paper there were about 30 participants per condition in the laboratory studies, the new experiments have a minimum of 70 participants per condition, thus providing much more highly powered tests.

**Table 2.** Effect sizes of the experiments in the current and original investigation demonstrating the effect of having people sign a veracity statement attesting to their honest reporting placed before versus after reporting

Study	Sample size	Number of conditions	Cheating task	Population	Average performance reported effect size (*d*) [95% CI]^*^
**This study**					
Study 1	444	6	Die rolling	Community laboratory	0.11 [−0.09, 0.30]
Study 2	408	4	Anagrams	Community laboratory	−0.01 [−0.20, 0.18]
Study 3	442	2	Anagrams	MTurk	0.05 [−0.14, 0.24]
Study 4	743	2^†^	Anagrams	MTurk	−0.05 [−0.19, 0.10]
Study 5	2,522	2	Anagrams	Naive MTurk	0.01 [−0.07, 0.09]
Study 6 (direct replication of PNAS study 1)	1,235	2	Paper matrix; self-reported travel expenses	Community laboratory	0.04 [−0.07, 0.15]^‡^
**Shu et al. (1) study**					
Study 1	101	3	Paper matrix; self-reported travel expenses	Students	−1.05 [−1.55, −0.53]^‡^
Study 2	60	2	Paper matrix; self-reported travel expenses	Students	−0.53 [−1.04, −0.01]^‡^
Study 3	13,488	2	Odometer reading reported on audit form	Automobile insurance clients	−0.20 [−0.16, −0.23]

* For all tasks, effect sizes are reported for the differences in total amounts reported between conditions. Negative effect size indicates reduction in cheating.

† This excludes a third “video” condition.

‡ Effect sizes reported in the last column are based on the paper matrix performance only, not the claimed travel expenses.

Whereas the original PNAS findings show that having the veracity statement that people are asked to sign at the beginning promotes honesty (1), in the new study 1, there was no difference in the average performance reporting across conditions. After rolling a 12-sided die twice, there was no significant difference between the average when it was reported when individuals were asked to sign first (*M* = 14.76) and when they were not asked to sign first^†^ (*M* = 14.48) (*t*[272] = −0.49, *P* = 0.62).

All conditions displayed cheating, considering the averages significantly differed from the expected average of the distribution (*M* = 13) (*P* < 0.001).^‡^ In other words, all groups had similar rates of relatively low cheating regardless of condition.

A limitation of this first new study is that there was no way to tell which individuals cheated. Therefore, studies 2–5 (*n* = 4,115) were designed to be able to detect cheating on the individual level. In those four new studies, people who were asked to sign the veracity statement before reporting were no more likely to cheat than people who were asked to sign the veracity statement after reporting (for percent of cheating *P* = [0.81, 0.82, 0.78, 0.91] or amount of cheating *P* = [0.76, 0.58, 0.52, 0.91]). Bayes *t* tests were run to obtain a Bayes factor (the default prior of an alternative effect size of *r* = 0.707 was used, which is consistent with the original paper).^§^ The Bayes factors ranged from 6.25 to 20.00. Thus, each study provides substantial to strong evidence in support of the null hypothesis (6).

In the direct replication study 6, we preregistered our methods and analytic plan (5) and had the power to detect an effect size of *d* = 0.10 at 80% power. We failed to detect an effect of signing first on all three preregistered outcomes (percent of people cheating per condition, one-sided *t* tests, *t*[1,232.8] = 1.25, *P* = 0.8942, *d* = 0.07 95% CI [−0.04, 0.19]; amount of cheating per condition, *t*[1,229.3] = −0.717, *P* = 0.7633, *d* = 0.04 95% CI[−0.07,0.15]; and amount of expenses reported, *t*[1,208.9] = −1.099, *P* = 0.864, *d* = 0.06 95% CI[−0.04, 0.17]). The Bayes factors for these three outcome measures were between 7.7 and 12.5, revealing substantial support for the null hypothesis (6). This laboratory experiment provides the strongest evidence to date that signing first does not encourage honest reporting.

Further suggestive evidence in favor of the null (of a negligible effect size) is the fact that Bayesian analysis reveals that 88% of the posterior density falls within the conventional region of practical equivalent (ROPE) of Cohen’s *d* of [−0.1, 0.1]. For this analysis, we set a very weakly informative prior (normal distribution with mean = 0 and SD = 10) (7). Drawing on previously published methodology and code (8, 9), we analyzed studies 1–5 sequentially and then used the final prior as the basis for analysis of study 6. We found the highest density interval (HDI) interval of the standard effect size estimate (for the percent of people cheating per condition) to be [−0.07, 0.14], and 88% of the posterior distribution falls within the ROPE. While not conclusively equivalent to zero, this provides further suggestive evidence for a negligible or nonexistent effect (for a discussion of decision rules using HDI + ROPE, see ref. 10).

**Fig. 1.**
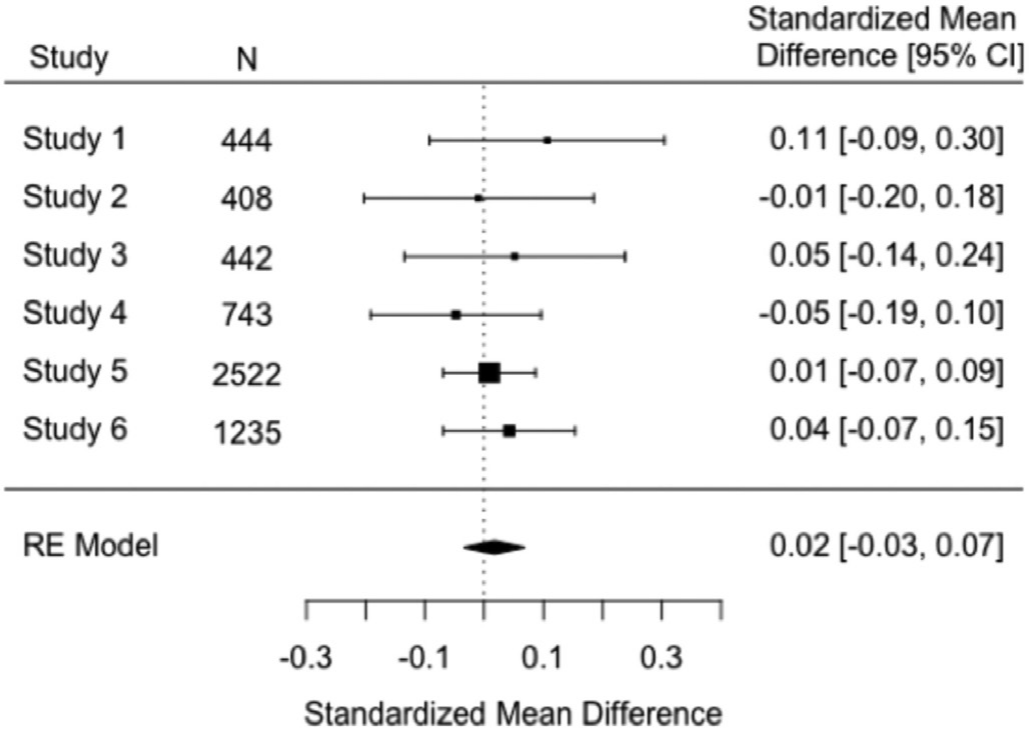
New studies: The effect of signing first on self-reported performance. Zero (illustrated with the dotted line) indicates no mean difference, the point corresponds to the point estimate for the effect size (Cohen’s *d*) for each individual study, and the error bars represent the 95% confidence interval around the effect size.

Fig. 1 shows a forest plot, depicting the effect sizes across the six replications, along with the metaanalytic effect size for amount reported and confidence intervals. Negative effect sizes indicate a reduction in cheating.

## Discussion

In the original PNAS paper, across two laboratory experiments (*n* = 161), the authors found that asking participants to sign a veracity statement placed at the top of a tax form increased honest responding as compared to at the end of the tax form (1). After conducting six studies that were larger and more highly powered (*n* = 5,794), including one direct replication of one of the two original PNAS laboratory studies, we did not replicate a sign-first effect.

Our studies contained a mix of online and laboratory experiments, and we used three different paradigms (anagram task, a die-rolling task, and a matrix task with all solvable matrices but strict time pressure). Our participants ranged from experienced Amazon MechanicalTurk (MTurk) workers to naive MTurk workers to university students to Boston and Chicago community members.** See Table 3 for a list of moderators we tested.

**Table 3.** Potential moderators we explored (and failed to detect) that we hypothesized could moderate the effect of having people sign a veracity statement attesting to their honest reporting placed before versus after reporting

Potential moderator	Relevant studies
Reporting online via typing vs. reporting on paper via handwriting	Electronic reporting vs. handwriting reporting (studies 1 and 2)
Study population	Laboratory population (Boston, studies 1,2, and 6; and Chicago, study 6; both community and student populations in Boston and Chicago) vs. MTurk (studies 3–5)
Verbal vs. written instructions	Study instructions written on computer screen/paper and participants read on their own (studies 1 and studies 3–5) vs. research assistant provided instructions out loud (studies 2 and 6)
Task type	Die rolling (study 1), anagram/word scramble (studies 2–5), matrix task (study 6), and expense reporting (study 6)
Incentive/amount of additional money at stake ($50 and under)	Raffle for $50 (study 1), up to $10 (study 2), $0.10 per reported answer (study 3 and 4), $0.30 per reported answer (5), and up to $42 for reported answer and reported expenses
Amount of baseline cheating	Study 3 (lowest cheating rate, 23% in the control group) to study 6 (highest cheating rate, 56% in the control group)
Type of reporting form	Regular participation form where generally there is no expectation of an honesty prompt (studies 1–5) vs. official-looking tax form where in naturalistic context there is a general expectation of an honesty prompt (study 6)

There are a number of explanations for why the original PNAS authors may have obtained positive results from the two laboratory experiments. It could have been type I error due to small sample sizes or some other problem in running the experiments. Another possibility is the fact that the study might have been conducted with insufficient supervision. Although the original authors had always trusted the data, given the new highly powered data, the original authors now question those original results.

The original authors have a third laboratory experiment, which was not included in the originally published PNAS paper, where signing first significantly reduced dishonesty; however, this was compared with no honesty statement or signature. While we have two examples (one from Shu et al. (1) and one from this paper [study 1]) failing to find a difference between signing at the bottom or no signature, we do not have enough evidence to make claims in the current paper about the effect of signing versus not signing at all. Therefore, we would like to emphasize that the focus of this paper is the effect of signing an honesty declaration at the top of a form compared to the bottom and not to make a claim regarding the effectiveness of priming honesty in general, be it through honor codes, oaths, or honesty statements.

In addition, in terms of the field experiment from the original PNAS paper, with the failure to directly replicate the results of the original laboratory experiment 1, we now look at the field study with new eyes and are concerned with its potential flaws, including the significantly different odometer readings at baseline. At baseline (before the study was conducted), individuals in the control group, on average, had driven 75,034.50 miles (SD = 50,265.35), while individuals in the treatment group had driven, on average, 59,692.71 miles (SD = 49,953.51), *t*_(13,474)_ = 17.78, *P* < 0.0001.

Before writing up this report, we searched the current literature (specifically, the papers citing the original finding), and a subset of the authors put out a call for academic papers in order to conduct a metaanalysis; however, we were unable to find any papers that looked explicitly at the effect of signing at the top of a form compared with signing at the bottom in the context of honesty (for example, we found papers using signing first as the control, papers about oaths more generally, or signing in a consumer behavior context). We also received no papers from other scholars that could help us in this attempt. For more information about our criteria for inclusion, the places we circulated this call for papers, and the papers we received, see *SI Appendix*. In this paper we tested numerous conceptually relevant moderators, and none of them influenced the effect. Therefore, we can conclude from these data that there is no good evidence for the existence of this effect, and the limited times it has been documented in the literature are likely a type 1 error.

We would like to emphasize that the focus of this paper is the replicability of the previously reported effect of signing an honesty declaration at the top of a form compared to the bottom. Consequently, instead of continuing to explore whether or when signing at the top versus the bottom reduces dishonesty, future research could focus on the underlying principles. For example, future research could examine whether other approaches to moral salience—such as stronger invocations of one’s identity—could reduce dishonesty, in light of our newest evidence that simply moving the signature box from the bottom to the top of a form is an insufficient intervention. What remains to be resolved is the necessary and sufficient conditions, if any, for the relevant manipulation to link subsequent behavior to conceptions of the self as honest and thus alter such behavior. This may depend on contextual factors not explored in the present research undertaking.

Small changes to the decision-making environment (“nudges”) have been shown to increase organ donation (12), retirement savings (13), and tax collection (14). The IRS estimates that over $387 billion are underreported annually, with over 68% of that money owed by individual taxpayers (15). Moving the veracity statement that people are asked to sign at the bottom to the front of the tax form has been touted as a “quick win.” However, even these seemingly simple changes can incur significant implementation costs. When one of the authors (Whillans) worked with a local government, they spent $15,000 of labor costs and 6 mo trying to implement this seemingly simple change.^††^ With the new data presented here, we recommend that practitioners take this finding out of their intervention “tool-kit” as it is unlikely to increase honesty.

## Materials and Methods

We obtained informed consent from all participants. We received ethical approval from the Institutional Review Boards of Harvard University (IRB18-1518), Boston University (#5010E), and the University of Chicago (IRB18-1420). For demographic characteristics of participants, see *SI Appendix*, Table S1. See https://osf.io/3javq/ for materials, implementation protocol, and all data and code.

### Study 1.

A sample of 444 adults was recruited from Harvard Business School’s Computer Lab for Experimental Research community pool to participate in the study in exchange for $2 and a chance of winning one of eight $50 prizes. Participants rolled a 12-sided die twice and wrote down the sum of the two rolls. The total reported corresponded to the number of entries into the raffle to win one of the eight prizes. Participants were randomly assigned to sign a veracity statement at the top of the form, before reporting; to sign at the bottom of the form, after reporting; or they did not sign an honesty declaration. Participants reported both online and on paper. See *SI Appendix*, Tables S2–S4, for more details.

### Study 2.

A sample of 408 adults from Harvard’s Decision Science Laboratory were recruited to participate in a study to earn up to $10. Participants were given a list of 10 scrambled words. They were given 3 min to unscramble as many words as they could. Participants were told they would be paid $1 for each word they reported unscrambling. Participants were asked to sign (or type) a veracity statement at the top of the form, before reporting, or at the bottom of the form, after reporting. Participants reported both online and on paper. See *SI Appendix*, Tables S5–S7, for more details.

### Study 3.

An online panel of 442 adults was recruited from Amazon MechanicalTurk (MTurk) to participate in a study in exchange for $0.50 and a potential bonus of up to $0.50. Participants completed the same task as described in study 3 on an electronic device, but were given 5 words to unscramble instead of 10, with a $0.10 bonus per answer. Participants were asked to sign a veracity statement electronically using their trackpad before or after reporting.

### Study 4.

An online panel of 912 adults was recruited from MTurk to participate in a study in exchange for $0.50 and a potential bonus of up to $3.50. Participants completed the same task as described in study 3, but were given seven words to unscramble instead of five, with a $0.50 bonus per answer reported. Participants signed a veracity statement using their trackpad before or after reporting. The first two conditions had 743 participants. A third condition was added, where participants had to report by submitting a video of themselves saying their name and how many they solved.

### Study 5.

An online panel of 2,522 adults was recruited from MTurk to participate in a study in exchange for $0.50 and a potential bonus of up to $2.10. Participation was limited to naïve MTurk participants, who completed fewer than 50 human intelligence tasks. This allowed us to rule out the possibility that participants may have heard of the intervention. Participants completed the same task as described in study 3, but were given $0.30 bonus per answer reported. Participants signed a veracity statement using their trackpad before or after reporting. Due to a typo in the survey software, participants read in the recruitment and consent form that the bonus would be $0.30 and that was the bonus paid out; however, on the actual screen where they were asked to report the number solved, it said in small text they would receive a $0.50 bonus. Since this was the same across conditions, we do not worry about the validity of our results, but this may have influenced the overall cheating rate.

### Study 6.

A sample of 1,235 adults was recruited across four laboratory sites (Harvard’s Decision Science Laboratory pool, Boston University Questrom School of Business community pool, and the University of Chicago Booth School of Business Center for Decision Research campus and downtown laboratories) to participate in a direct replication of study 1 from Shu et al. (4) excluding the pure control condition. Participants completed a matrix task and then filled out a tax form where they reported the income from the matrices task, as well as their commuting expenses. Participants were paid a $2 show-up fee and had the potential to earn up to an additional $40 depending on reported number of matrices solved and commuting expenses. The results reported above hold controlling for testing location. See *SI Appendix*, Table S8 for more details.

## Supplementary Material

Appendix (PDF)

The SI Appendix has been updated to correct Tables S5-S7.

## Data Availability

Data deposition: Materials, implementation protocol, and all data and code have been deposited at https://osf.io/3javq/.

## Footnotes

*The key results of these studies hold controlling for whether people signed a veracity statement in an online versus an offline environment.

^†^Given the lack of statistical significance between signing after and no signature in both Shu et al. (1) and in this study, we collapse across those two conditions to test signing first versus not signing first.

^‡^We tested this by running simulations in R and comparing our observed data distribution to that of 1,000,000 simulated die rolls.

^§^A Bayes *t* test provides a Bayes factor which assesses the strength of the evidence in favor of one hypothesis (the null) over the alternative.

**Because previous research has shown differences between different types of signatures (11), we used handwritten on paper, online typed, and online trackpad signatures. None of these features affected our key results: being asked to sign at the top of a form did not reduce dishonesty.

^††^This effort was before the replications were conducted and failed for other administrative reasons.
