# Supplementary material for: Signing at the beginning versus at the end does not decrease dishonesty
Source: Proc Natl Acad Sci U S A. 2020 Mar 16;117(13):7103–7. doi: 10.1073/pnas.1911695117 (PMC7132248; doi:10.1073/pnas.1911695117)
Supplement: Supplementary File [file pnas.1911695117.sapp.pdf]

PNAS
www.pnas.org

Supplementary Information for

Signing at the beginning versus at the end does not decrease dishonesty

Authors: Ariella Kristal,[a] Ashley Whillans,[a] Max Bazerman,[a] Francesca Gino,[a] Lisa Shu,[b] Nina Mazar,[c] & Dan Ariely[d]

Corresponding Author: Francesca Gino
Email: fgino@hbs.edu

This PDF file includes:

Supplementary text
Tables S1 to S9
SI References

www.pnas.org/cgi/doi/10.1073/pnas.1911695117

Supplementary Information Text

Study 1
Methods. Four-hundred and forty-four participants were asked to fill out a number of surveys in a computer lab as part of a larger set of studies. We followed a similar methodology as laid out by Chou (1). For all participants, the first survey page asked participants to: "Please roll the green die in front of you. On the next page, you will be asked to record the numbers that came up on the first two rolls. That total number will be the number of entries you will receive in a raffle to win one of three $50 prizes. You may roll the die as many times as you would like to check that it is a fair die, but please record only the numbers from the first two rolls." All participants were then randomly assigned to one of six conditions where they were asked to report the sum of their dice rolls. Half of the participants were assigned to report their total rolls electronically on a survey form and the other half were assigned to report their total on a paper survey form. All participants saw the instructions "Please enter [write] the numbers you rolled to indicate how many raffle tickets you should receive. Every additional raffle ticket you receive will increase your chances of winning one of three $50 prizes." In both the electronic and paper and pencil reporting setting, there were three conditions: participants were not asked to sign a veracity statement (control condition), participants were asked to sign a veracity statement at the top of the survey form (before reporting their total), or participants were asked to sign a veracity statement at the bottom of the survey form (after reporting their total). The signature prompt was "Please provide your signature to certify that the information you will provide is valid, by typing [signing] your name below."

Results. Tables S2-S4 below provide the summary statistics from Study 1 and the various configurations of the different conditions. There was no significant difference between the total reported across the six conditions $F_{(5,438)}$ =0.663, p = 0.65. The expected value across the roles is 13 and all 6 conditions are significantly different from the simulated expected value, suggestive of a small but significant amount of dishonest reporting in each of the conditions (See https://osf.io/3javq/ for simulations).

Study 2
Methods. Four-hundred and eight participants recruited in the lab were given a laminated sheet of paper with 10 scrambled words listed and were told they would have three minutes to try to unscramble as many as they could. The words are from Aspin and Richter (3). After three minutes, participants were asked to report the number of anagrams that they solved and were given a $1 bonus for each anagram they reported to have solved. Participants were randomly assigned to either report the total solved electronically in a survey form or a paper survey form. All participants read, "How many anagrams were you able to unscramble? Please select [write] the total number below, but wait to submit until the research assistant returns. You will then be paid $1 for each answer your report being able to solve." Within the two reporting formats (electronic and paper/pencil), participants were randomly assigned to either sign a veracity statement at the top of the survey form (before reporting) or at the bottom of the survey form (after reporting). Participants were prompted with the sentence "Please type [provide] your signature to certify that the information you provided is valid."

Results. Only three of the ten anagrams were solvable, so any participant who reported more than three solved was definitely cheating. However, the study design did not allow to identify the exact words people did or did not lie about solving. Tables S5-S7, below, show the summary statistics from Study 2 and the various configurations of the different conditions. There was no significant difference between the four conditions with respect to the percent of participants reported to having solved more than three anagrams $F_{(3,407)}$ = 0.121, p = 0.95 or in the total number reported solved, $F_{(3,404)}$ = 0.39, p = 0.76.

Study 3
Methods. Four-hundred and forty-two Amazon Mechanical Turk workers were first presented with a list of five of the ten scrambled words used in Study 2. We reduced the full list to five to see

if we could detect a difference with lower variance. All participants were then given three minutes to report the number of words that they were able to unscramble. Participants were randomly assigned to sign a veracity statement using their mouse or tracking pad before or after reporting the total they solved. All participants saw the prompt "Please sign below to certify that you are going to truthfully report [truthfully reported] the number of words you were able to unscramble. (To sign, please use a laptop trackpad, a mouse, or your finger on a touchscreen)" Participants were paid a $0.10 bonus for each anagram they reported solving.

Results. In the sign first condition, 24% of participants reported more than 3 anagrams solved, i.e. cheated ($M_{reported}$ = 3.21, $SD_{reported}$ = 0.95), and in the sign last condition, 23% of participants cheated ($M_{reported}$ = 3.16, $SD_{reported}$ = 0.98). There was no significant difference between the two conditions with respect to percent of cheaters $t_{(440)}$= -0.23, p = 0.82 or in amount of reported anagrams solved $t_{(439)}$= -0.56, p = 0.58.

Study 4
Methods. Nine-hundred and twelve participants on MTurk who passed an attention check were presented with a list of seven anagrams. Only three were solvable. We introduced an attention check at this point to see if the lack of attention check could account for our inability to detect an effect of the signature timing in the previous studies. After three minutes, all participants were asked to report the number of anagrams that they solved and they were given a $0.10 bonus for each anagram they reported solving. Participants were randomly assigned to one of three conditions: the first two conditions were the same sign first and sign last conditions from Study 3, described above, and in the third condition, participants uploaded a video of themselves reporting the number they solved. Participants in the first two conditions were prompted with "Please sign below to certify that you are going to truthfully report [truthfully reported] the number of words you were able to unscramble. (To sign, please use a laptop trackpad, a mouse, or your finger on a touchscreen)." Participants in the video condition were instructed "Please record a video of yourself saying your name and the number of anagrams you were able to unscramble. Then upload the file below."

Results. In the sign first condition, 41% of participants reported more than three anagrams solved, i.e. cheated ($M_{reported}$ = 3.54, $SD_{reported}$ = 1.33), in the sign last condition, 40% of participants cheated ($M_{reported}$ = 3.61, $SD_{reported}$ = 1.59), and in the video last condition 38% of participants cheated ($M_{reported}$= 3.46, $SD_{reported}$ = 1.33). While the results in the main text only consider the participants in the first two conditions (sign first and last), here we include the video last condition in our analysis. Once again there were no significant differences between the three conditions with respect to the percent of cheaters $F_{(2,909)}$ = 0.28, p = 0.76 or the amount of reported anagrams solved $F_{(2,909)}$ = 0.68, p = 0.51.

Study 5
Methods. In this pre-registered study, 2,522 naive participants on MTurk (who had fewer than 50 HITs approved) who passed an attention check were presented with a list of seven anagrams. Only three were solvable. After three minutes, all participants were asked to report the number they solved and were given a $0.30 bonus for each anagram they reported to have solved. Participants were randomly assigned to either sign before or after reporting the number they solved. Participants were prompted with the same instructions described in studies 3 and 4. The sample size was chosen to be powered to detect a small effect size (d = 0.1).

Results. In the sign first condition, 28% of participants reported more than three anagrams solved, i.e. cheated ($M_{reported}$ = 3.15, $SD_{reported}$ = 1.11), and in the sign last condition, 28% of participants cheated ($M_{reported}$ = 3.14, $SD_{reported}$ = 1.18). There was no significant difference between the two conditions with respect to the percent of cheating $t_{(2520)}$= -0.11, p = 0.91 or in amount of reported anagrams solved. $t_{(2511)}$= -0.11, p = 0.91.

Study 6

Methods. In this pre-registered direct-replication study, 1,235 participants were brought into one of four different labs (one in Cambridge, MA, one in Boston, MA, and two in Chicago, IL). We followed the exact protocol of Experiment 1 (excluding the "no signature" control condition) outlined in the 2012 PNAS paper (1) and used the original materials, as well. As described in Shu et al. (1):

> "*For this task, participants received a worksheet with 20 math puzzles, each consisting of 12 three-digit numbers (e.g., 4.78) and a collection slip on which participants later reported their performance in this part of the experiment. Participants were told that they would have 5 min to find two numbers in each puzzle that summed to 10. For each pair of numbers correctly identified, they would receive $1, for a maximum payment of $20. Once the 5 min were over, the experimenter asked participants to count the number of correctly solved puzzles, note that number on the collection slip, and then submit both the test sheet and the collection slip to the experimenter. We assume respondents had no problems adding 2 numbers to 10, which means they should have been able to identify how many math puzzles they had solved correctly without requiring a solution sheet. Neither of the two forms (math puzzles test sheet and collection slip) had any information on it that could identify the participants. The sole purpose of the collection slip was for the participants themselves to learn how many puzzles in total they had solved correctly. Tax return form. After the problem-solving task, participants went to a second room to fill out a research study tax return form (based on IRS Form 1040). The one-page form we used was based on a typical tax return form. We varied whether participants were asked to sign the form and if so, whether at the top or bottom of the page. Participants filled out the form by self reporting their income (i.e., their performance on the math puzzles task) on which they paid a 20% tax (i.e., $0.20 for every dollar earned). In addition, they indicated how many minutes it took them to travel to the laboratory, and their cost of commute. These expenses were "credited" to their post-tax earnings from the problem-solving task to compute their final payment. The instructions read: "We would like to compensate participants for extra expenses they have incurred to participate in this session." We reimbursed the time to travel to the laboratory at $0.10 per minute (up to 2 h or $12) and the cost of participants' commute (up to $12). All of the instructions and dependent measures appeared on one page to ensure that participants knew from the outset that a signature would be required. Thus, any differences in reporting could be attributed to the location of the signature*"

Results. We failed to detect an effect of the sign first condition on all three pre-registered outcomes (percent of people cheating per condition, $t(1232.8) = -1.50$, $p = 0.8942$, $d = -0.07$ 95%CI[-1.96, 0.976]; amount of cheating per condition, $t(1229.3) = -0.717$, $p = 0.7633$, , $d = -0.04$ 95%CI[-1.96, 0.976]; and amount of expenses reported, $t(1208.9) = -1.099$, $p = 0.864$), , $d = -0.06$ 95%CI[-1.96, 0.976]. See below for the summary statistics by location.

Meta-analysis: Call for Papers

In September 2018, we put out the following call for papers for a meta-analysis of the signing first effect: [1]

> "Dear colleagues,
>
> We are conducting a meta-analysis comparing signing before reporting an outcome vs. signing after (or not signing at all) and the effect on honesty. We are looking for published or unpublished empirical studies that satisfy the following criteria:
>
> 1. <u>Signing Manipulation:</u> The main manipulation must be a signing intervention where one of the conditions signed before reporting an outcome and there must be a control

[1] Note that while the call includes papers with signature before versus no signature at all, for the purpose of this paper, we only consider signing first vs signature last.

condition of either no signing OR signing after reporting. If you have a signing study and you are not sure if it is eligible, please send it along.

2. Honesty: While our primary outcome of interest is honesty, if your study satisfies the signing manipulation criteria but the outcome is in a different domain (e.g. commitment), please send it across as well.

3. Language: The studies must be in English, or have an English translation so we can understand the study design and extract effect sizes.

If you have a published or unpublished manuscript, dissertation chapter or a poster that satisfies these criteria, please send it to Ariella Kristal (akristal@hbs.edu). We will review your contribution, and may ask for further information needed to extract effect sizes. The process should involve little additional effort on your end."

We emailed the following listservs: NYU Ethics, Society for Judgment and Decision-Making, Society for Personality and Social Psychology, Society for Experimental Social Psychology, Academy of Management: Organizational Behavior Division, Academy of Management: Organizational Behavior Division, Organization and Management Theory Division, Academy of Management: COGNET, Journal of Behavioral Public Administration, Behavioral Science and Policy, Behavioral Public Policy, and the Behavior Change for Good listserv.

We only received three papers in response (4-6). One paper provides people with the option to sign an oath and then measures dishonesty, and the two other papers did not have honesty dependent variables.

Table S1. Demographic information for participants in Studies 1-6

Study	Population	% Male	Age (mean)	Age (sd)
1	Community lab	51.87	34.84	14.50
2	Community lab	45.67	30.04	12.51
3	Mturk	57	NA	NA
4	Mturk	49	NA	NA
5	Naïve Mturk	42	NA	NA
6	Community lab	49.79	31.26	13.77

*NA denotes that corresponding data was not collected.

Table S2. Summary statistics by condition (Study 1)

Condition	DV: Reported sum of two 12-sided die rolls		N
	Mean	SD	
No signature - paper	15.42	4.55	76
Sign first - paper	14.74	5.85	73
Sign last - paper	14.12	5.29	73
No signature - electronic	14.15	5.83	75
Sign first - electronic	14.78	5.38	72
Sign last - electronic	14.23	5.47	75

Table S3. Summary statistics by signature condition, DV = Reported sum of two 12-sided die rolls (Study 1)

Condition	Mean	SD	N
No signature	14.79	5.25	151
Sign first	14.76	5.60	145
Sign last	14.18	5.37	148

Table S4. Summary statistics by medium condition, DV = Reported sum of two 12-sided die rolls[2]

Condition	Mean	SD	N
Paper	14.77	5.25	222
Electronic	14.38	5.55	222

[2] These results also contradict the results of Studies 1 and 2 in Chou (2015 (2))

Table S5. Summary statistics, DV = Reported number of anagrams solved by condition (Study 2)

Condition	Mean	SD	N	Percent who cheated
Sign first. - paper	4.574	2.260	101	57%
sign last - paper	4.359	2.169	103	55%
Sign first - electronic	4.267	2.019	101	55%
Sign last - electronic	4.500	2.114	102	59%

Table S6. Summary statistics, DV = Reported number of anagrams solved by signature condition (Study 2)

Condition	Mean	SD	N	Percent who cheated
Sign first	4.421	2.143	202	56%
Sign last	4.429	2.138	205	57%

Table S7. Summary statistics, DV = Reported number of anagrams solved by medium condition (Study 2)

Condition	Mean	SD	N	Percent who cheated
Paper	4.466	2.211	204	56%
Electronic	4.384	2.066	203	57%

Table S8. Summary by condition and location (Study 6)

Location	Boston University				UChicago Campus Lab				UChicago Downtown Lab				Harvard University			
Condition	N	% cheat	# Over reported (SD)	Expenses (SD)	N	% cheat	# Over reported (SD)	Expenses (SD)	N	% cheat	# Over reported (SD)	Expenses (SD)	N	% cheat	# Over reported (SD)	Expenses (SD)
Sign First	46	54%	1.91 (3.26)	5.99 (4.95)	39	56%	3.54 (7.36)	7.08 (7.88)	77	74%	4.70 (5.48)	9.13 (5.67)	448	58%	2.73 (4.45)	7.30 (7.77)
Sign Last	54	56%	2.67 (5.06)	6.82 (6.36)	39	56%	3.69 (6.14)	6.85 (11.06)	77	78%	5.77 (6.20)	10.86 (5.30)	455	53%	2.20 (3.90)	6.34 (6.27)

Table S9. The signature prompt for each of the six new studies. The location of the prompt was before [or after] the participant provided a self-report.

Study	Signing prompt
1	Please provide your signature to certify that the information you will provide [provided] is valid, by typing [signing] your name below
2	Please type [provide] your signature to certify that the information you will provide [provided] is valid.
3	Please sign below to certify that you are going to truthfully report [truthfully reported] the number of words you were able to unscramble.
4	Please sign below to certify that you are going to truthfully report [truthfully reported] the number of words you were able to unscramble.
5	Please sign below to certify that you are going to truthfully report [truthfully reported] the number of words you were able to unscramble.
6	I declare that I carefully examined this return and that to the best of my knowledge and belief it is correct and complete.

References

1. L. Shu, N. Mazar, F. Gino, M. D. Ariely, M. Bazerman, Signing at the beginning makes ethics salient and decreases dishonest self-reports in comparison to signing at the end. *Proc. Natl. Acad. Sci. U.S.A. 109*(38) 15197-15200, (2012).
2. E. Chou, What's in a name? The toll e-signatures take on individual honesty. *J. Experimental Soc. Psychol.*, *61*, 84-95 (2015).
3. L. Aspinwall, L. Richter, Optimism and self-mastery predict more rapid disengagement from unsolvable tasks in the presence of alternatives. *Motivation and Emotion,* 23, 221-245 (1999).
4. N. Jacquemet, S. Luchini, A. Malezieux, J. Shogren, Who'll stop lying under oath? Empirical evidence from Tax Evasion Games. halshs-02159905f (2019). Available at https://halshs.archives-ouvertes.fr/halshs-02159905/document
5. K. Kettle, G. Häubl, The signature effect: Signing influences consumption-related behavior by priming self-identity. *J. Consumer Res.*, 38(3), 474-489 (2011).
6. A. Chin, D. Beckett, Don't watch me read: How mere presence and mandatory waiting periods affect consumer attention to disclosures. *Behavioural Public Policy*, 1-20 (2018).
